# Brain Oscillations and Autonomic Synthonization via Comodulation in Collaborative Negotiation

**DOI:** 10.3390/e27080873

**Published:** 2025-08-18

**Authors:** Katia Rovelli, Carlotta Acconito, Laura Angioletti, Michela Balconi

**Affiliations:** 1 International Research Center for Cognitive Applied Neuroscience (IrcCAN), Università Cattolica del Sacro Cuore, Largo Gemelli 1, 20123 Milan, Italymichela.balconi@unicatt.it (M.B.); 2 Research Unit in Affective and Social Neuroscience, Department of Psychology, Università Cattolica del Sacro Cuore, 20123 Milan, Italy

**Keywords:** neural synthonization, negotiation, decision-making, emotion regulation, autonomic responses, inter-brain connectivity

## Abstract

This study investigates the relationship between neural and physiological synthonization via comodulation (Synth) in dyadic exchanges centered on negotiation processes. In total, 13 dyads participated in a negotiation task with three phases: Initiation (IP), Negotiation Core (NCP), and Resolution (RP). Electroencephalographic (EEG) frequency bands (i.e., delta, theta, alpha) and autonomic responses (heart rate variability, HRV) were recorded. Synth was analyzed using Euclidean distance (EuDist) for EEG and autonomic indices. Significant Synth in delta, theta, and alpha bands in temporo-central and parieto-occipital regions was observed, indicating social cognitive alignment. HRV Synth was higher during the NCP than IP, suggesting better coordination. Based on this result, a cluster analysis was performed on HRV EuDist to identify distinct groups based on HRV, and eventually personality patterns, that revealed one cluster with higher Synth and reward sensitivity, and another with lower Synth and reward sensitivity. These findings show how neural and autonomic Synth enhances social cognition and emotional regulation.

## 1. Introduction

Negotiation is a fundamental process in human interaction, particularly in contexts requiring cooperation to achieve a common goal [1,2]. While traditional models have predominantly focused on the cognitive aspects of negotiation, recent advancements in social neuroscience underscore the importance of considering the social dimensions of the negotiation process. It has become increasingly evident that negotiation cannot be fully understood by cognitive processes alone, as affective, embodied, and social factors also play a critical role in shaping outcomes [3,4]. In dyadic negotiation, where two individuals engage in reciprocal exchanges, the primary aim is to reach a shared decision that reflects mutual interests and aligns with the goals of both parties [5]. In such contexts, negotiation extends beyond mere compromise and delves into the creation of a joint decision that incorporates the concerns and perspectives of both individuals. In this framework, the role of synthonization via comodulation and emotional dynamics in social interactions, or “Synth”, becomes particularly relevant, influencing both decision-making and the ability to adapt behavior in real-time. In this context, individual differences in personality and decision-making are crucial factors [6,7,8]. These differences significantly shape how individuals approach negotiations, guiding their decision-making processes and influencing their behaviors in interpersonal interactions. Theoretical models suggest that personality traits influence negotiation strategies by affecting risk perception, adaptability, and interpersonal sensitivity [6,7,8].

For instance, dual-process theories of decision-making [9] distinguish between intuitive and deliberative reasoning, which align with individual differences in cognitive styles. Similarly, trait-based models, such as the Five-Factor Model [10], highlight how characteristics like agreeableness and emotional stability predict cooperative versus competitive negotiation behaviors. The reinforcement sensitivity theory further posits that variations in reward and punishment sensitivity shape approach and avoidance tendencies in social contexts.

Furthermore, a first strand of cognitively oriented theories has sought to explain how negotiation emerges as a structured process that balances individual and collective interests. However, to fully capture the dynamics of negotiation, it is essential to include social interaction and Synth between parties. Traditional models emphasize the relationship between decision-making strategies and cooperative problem-solving, positing that successful negotiation arises from the ability to integrate self-interest with mutual benefit [11,12]. Within this framework, the dual-concern model [13,14] emphasizes the balance between self-interest and concern for others, shaping negotiation strategies. Similarly, the cooperative-competitive framework [15,16] highlights the interplay between cooperation and competition, influencing negotiation dynamics and outcomes.

More recent models, such as the Shared Intentionality Theory [1], expand on these frameworks by emphasizing the importance of joint action and mutual understanding. This theory suggests that humans have evolved cognitive mechanisms specifically designed to enable collaboration, which are crucial in negotiation scenarios that require alignment of goals and intentions [17]. In the context of dyadic negotiation within a group setting, the need for shared understanding takes on additional complexity, as the decision made by the dyad may need to reflect how the group should act, requiring alignment not only between the two negotiators but also with broader group norms and expectations. Indeed, the cognitive dimension of negotiation is further reinforced by the role of perspective-taking and shared mental models [18]. The construction of common ground and shared representations plays a pivotal role in facilitating coordination, as it allows dyads to predict and adapt to each other’s responses [19].

In this context, hyperscanning studies have provided compelling evidence that neural Synth, particularly in the lower-frequency bands, plays a critical role in fostering cooperation and shared intentionality [20,21]. These findings suggest that Synth in social decision-making is not merely a reactive phenomenon, but reflects an anticipatory alignment of internal states across interacting partners. In line with recent models of predictive coding and active inference, negotiation can be conceptualized as a dynamic process of reciprocal model alignment, whereby agents engage in mutual prediction and error minimization to reduce uncertainty in social interaction [22]. From this perspective, inter-brain Synth—especially in low-frequency bands such as delta and theta—may reflect the coupling of hierarchical generative models that aim to represent and anticipate the partner’s communicative intentions and behavioral responses. Furthermore, autonomic Synth, and particularly heart rate variability (HRV) coherence, may index the alignment of interoceptive predictions and emotional regulation processes, which are essential for adaptive interpersonal engagement. Such predictive processes operate across cortical and subcortical levels and facilitate the emergence of joint intentionality and shared decision-making through recursive cycles of expectation and correction.

Furthermore, research indicates that in cooperative negotiation, rather than relying on high-frequency oscillations linked to executive control, individuals exhibit increased delta- and theta-band power, reflecting a shift toward automatic and embodied decision-making strategies [23,24]. Lower-frequency oscillations have been linked to emotional attunement and embodied cognition, facilitating real-time adaptation to social cues [25,26]. These oscillatory patterns are thought to support attentional focus, emotional regulation, and sensory integration, which are essential for effective negotiation [27,28]. This interaction between lower-frequency oscillations and cognitive–emotional processes underscores their fundamental role in social interactions. Specifically, these rhythms not only enhance attentional focus and emotional regulation but also create a neurophysiological framework for integrating external social cues with internal predictive models. By modulating the balance between sensory-driven and cognitively controlled processes, these oscillations enable individuals to flexibly adjust their strategies during negotiation. Importantly, delta-band activity has been associated with integrating bottom-up sensory inputs with top-down cognitive control, facilitating a continuous exchange between external stimuli and internal cognitive frameworks [29,30]. At the same time, theta oscillations have been shown to support key functions such as memory encoding, prediction processing, and heightened sensitivity to social cues, which are crucial for anticipating and interpreting conversational dynamics [29]. Building on this, individuals engaged in negotiation rely on these frequency patterns to dynamically regulate their communicative strategies. By processing sensory inputs through delta activity and leveraging theta oscillations for predictive and mnemonic functions, negotiators can anticipate interlocutors’ responses, adjust their discourse in real time, and maintain alignment with shared goals [31]. Moreover, alpha activity also plays a crucial role in selectively filtering information and maintaining attentional control, essential components of shared decision-making. In this context, neural mechanisms must accommodate the shared cognitive load and Synth required to align the dyad’s responses with the broader group’s expectations and norms. This process not only necessitates the coordination of lower-frequency oscillations but also depends on alpha-mediated functions. Indeed, the literature emphasizes how these oscillations are thought to facilitate selective attention, allowing individuals to filter out irrelevant stimuli and focus on task-relevant cues [32]. Alpha waves have been observed to decrease during attentional engagement, particularly in contexts requiring focused cognitive processing [33]. This reduction in alpha power reflects an active suppression of distractors, directing cognitive resources toward social and emotional cues that are critical in the negotiation process. Moreover, the interaction of alpha with other networks, such as the dorsal attention network and the default mode network (DMN), highlights the balance between internally and externally directed attention during social decision-making [34]. This dynamic interrelation between networks is crucial for maintaining the adaptability and responsiveness required for successful negotiations.

Expanding on the previous discussion of neural Synth in negotiation, it is crucial to examine the specific brain networks involved in social cognition. The social brain network is key to processing social cues, supporting the alignment of individual actions with group expectations. Specifically, this network [35], which includes regions such as the superior temporal sulcus (STS), medial prefrontal cortex (mPFC), and temporoparietal junction (TPJ), is essential for processing social cues and attributing intentions. Activation in these regions facilitates perspective-taking and behavioral regulation, ensuring that negotiators remain attuned to each other’s mental states [36]. Notably, studies have found that theta oscillations in the mPFC and STS predict successful alignment in negotiation tasks [5,37], further emphasizing the role of low-frequency Synth in social cognition [38].

The role of neural Synth is further complemented by autonomic Synth, which contributes to mutual regulation and trust-building in dyadic interactions [39]. Moreover, research has indicated that autonomic coupling enhances interpersonal coordination by promoting non-verbal alignment and fostering a sense of shared emotional experience, which is particularly relevant in high-stakes negotiation scenarios [40]. In particular, the literature highlights the primarily role of cardiovascular components and galvanic skin response (GSR) as modulators of Synth in didactic cooperation and negotiation processes, focusing on physiological components such as heart rate (HR), HRV, skin conductance level (SCL), and skin conductance response (SCR) [41]. HR provides a broad measure of physiological arousal and engagement [42,43], while HRV captures fluctuations in autonomic balance, reflecting adaptability to effortful situation and social demands [44]. On the other hand, SCL and SCR offer insights into sympathetic nervous system activity, indicating emotional arousal and cognitive effort during decision-making processes [45]. Research suggests that autonomic responses dynamically evolve throughout the negotiation process, with HRV and SCL demonstrating the most extended temporal changes in response to cognitive and affective demands [46,47]. Specifically, SCL variations reflect attentional engagement and emotional intensity, marking critical moments of decision conflict and resolution [48]. Similarly, HRV has been extensively linked to emotion regulation, social engagement, and cognitive flexibility [39,49]. More specifically, the literature highlights how higher HRV coherence is associated with increased resilience and adaptability in social interactions, facilitating trust-building and cooperative negotiation strategies [40]. Therefore, these autonomic markers provide a window into the underlying regulatory processes that influence interpersonal dynamics, highlighting the relationship between physiological and cognitive mechanisms in achieving negotiation success.

However, no study has yet investigated the dynamic relationship between neural and autonomic Synth in the context of dyadic exchanges centered on negotiation processes. These interactions entail the implementation of a shared decision-making choice concerning the group, with a specific emphasis on the social dynamics. Specifically, to achieve this objective, the present study adopted the innovative hyperscanning paradigm, overcoming the limitation of single-brain detection and enabling the exploration of neural and physiological Synth within the dyad.

Building on existing theoretical frameworks, we hypothesize that specific neural and physiological patterns will emerge in response to the collaborative nature of the task. First, we expect enhanced neural Synth in the lower-frequency bands, particularly delta and theta, during the entirety of the negotiation process. Indeed, as previously mentioned, these frequency bands have been shown to play a role in facilitating interpersonal attunement and integrating sensory input with internal representations [29,30]. This Synth is likely to occur in brain regions that are involved in social cognition and attentional control, such as the temporo-central and parieto-occipital areas compared to the frontal area.

Secondly, given the cognitive demands of cooperative decision-making, we anticipate that individuals engaged in negotiation will exhibit increased alpha coherence in regions linked to attentional control and stimulus filtering [32,33]. Thus, we expect that dyads will exhibit higher Synth, particularly in the temporo-central regions, reflecting attentional modulation and the inhibition of task-irrelevant stimuli.

Thirdly, we predict that dyads exhibiting higher autonomic Synth, as measured by HRV Synth, will demonstrate greater success in building consensus during the negotiation. Autonomic coherence is a well-established indicator of social engagement and emotional regulation. Previous research, as previously mentioned, has shown that when individuals experience greater autonomic Synth, they tend to have more effective interactions, characterized by mutual understanding and cooperation [39,40]. Consequently, we hypothesize that dyads with stronger HRV Synth will engage in more collaborative negotiation processes and reach agreements more effectively.

Finally, following this hypothesis, it may also be conceivable to classify different dyads into distinct groups based on specific HRV patterns. Given that individual differences play a crucial role in physiological and neural responses, it is plausible that these individual traits also contribute to the formation of distinct clusters of dyads, each characterized by specific HRV patterns. Based on the previously mentioned literature, individuals with higher sensitivity to reward are more likely to engage in behaviors that promote social cooperation and mutual benefit. We predict that this heightened responsiveness to rewarding outcomes may enhance individuals’ capacity to align both neural and physiological states with their negotiation partners, particularly in contexts that offer potential rewards such as collaborative agreement or mutual benefit. Individuals with higher reward sensitivity might exhibit stronger Synth in neural circuits involved in motivation and reward processing as well as greater autonomic coherence, reflecting their heightened engagement in the negotiation process. Consequently, individuals with varying reward sensitivity levels may form distinct clusters, characterized by different patterns of HRV Synth, contributing to variability in negotiation success and collaborative outcomes.

## 2. Materials and Methods

### 2.1. Sample

A sample of 26 university students’, divided into 13 same-gender dyads, participated in the present study, which utilized a non-probabilistic convenience sampling approach for recruitment. A priori power analysis was performed using G*Power (version 3.1.9.7) to determine the minimum required sample size for a repeated-measures ANOVA with two groups and two time points. Assuming a medium effect size (f = 0.38), a significance level of 0.05, and a power of 0.95, the analysis indicated that a minimum of 26 participants would be sufficient. These parameters support the adequacy of our final sample (N = 26; 13 dyads).

Participants of each dyad were assigned to one of two experimental roles, designated as Member 1 and Member 2, through randomization (Member 1—Mean_age_ = 26.9, SD_age_ = 8.2; Member 2—Mean_age_ = 23.9, SD_age_ = 3.1). There was no prior acquaintance between the members of each dyad, thereby reducing the risk of biases or confounding variables that could influence the experimental outcomes. Participation in the study was voluntary, and no financial compensation or alternative rewards were offered. All participants were required to be right-handed and have normal or corrected-to-normal visual acuity. Individuals with a history of psychiatric or neurological disorders, significant depressive symptoms, or impaired cognitive functioning were excluded from this study. Additionally, participants with deficits in short- or long-term memory or those using psychoactive drugs known to affect cognitive processes were not eligible, ensuring that the results would not be compromised by these potential confounders. Prior to the beginning of the experiment, informed consent was obtained from all participants.

Ethical approval for this research was granted by the Ethics Committee of the Department of Psychology, Catholic University of the Sacred Heart, Milan, Italy. The study was conducted in full compliance with the ethical principles outlined in the Declaration of Helsinki (2013) and in accordance with the General Data Protection Regulation (GDPR, EU Reg. 2016/679).

### 2.2. Exerimental Procedure

**Figure 1:**
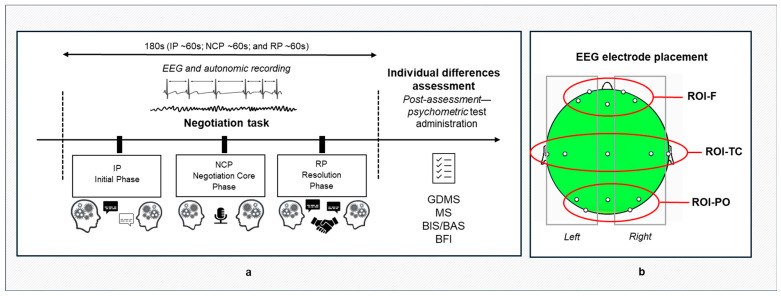
(**a**) Experimental procedure and (**b**) EEG electrode placement.

The experiment followed a structured protocol conducted in a controlled environment and lasted approximately 30 min (Figure 1). Participants were seated in a room with moderate lighting to allow natural interaction while minimizing external interferences. Autonomic and neural activity was simultaneously recorded from dyads using a hyperscanning paradigm, collecting data during both baseline resting conditions and the active negotiation phases.

#### 2.2.1. Negotiation Task

In the negotiation task, participants were guided to maintain a relaxed and still posture, speak clearly without whispering, and take turns to avoid overlapping conversations. The task involved a realistic scenario where participants had to decide how their actual workgroup would handle a situation in which a member did not align with the group’s ideals and working style (scenario: “*A member of your workgroup does not align with the group’s ideals and working style. What actions would your real workgroup take in such a situation?*”). To facilitate this decision-making process, participants were seated in front of a screen that displayed eight statements. Each statement outlined a potential strategy their real workgroup might adopt in response to the scenario (e.g., “*Reliable sources are consulted, and the decision is made accordingly*”; “*The opinion of the most experienced member of the group is followed*”; “*The group disbands, with some members insisting on one direction and others on another*”; etc.).

Each participant independently selected the statement they believed best represented their workgroup and read their choice aloud to the other member of the dyad. The experimenter recorded each participant’s initial selection as *Member 1’s initial choice* and *Member 2’s initial choice*.

Subsequently, participants engaged in a three-minute timed negotiation, during which they were required to collaboratively decide on a single statement that best reflected their shared perception of their real workgroup. The experimenter recorded this final agreed-upon statement as the dyad’s *final negotiated choice*. All dyads finished the negotiation process within the given time by reaching a common agreement.

The interaction process was identified as comprising three main phases based on the applied semantic criterion: Initiation Phase (IP), Negotiation Core Phase (NCP), and Resolution Phase (RP). The semantic criterion used for classification relies on the distinct linguistic functions and communication goals observed during each phase. The IP corresponds to the individual selection and verbalization of statements, with participants presenting their initial choices without interaction, focusing on expressing personal perspectives. The NCP involves a collaborative process where the dyad negotiates, compares, and adjusts their initial statements, working towards consensus. This phase is marked by active dialogue, questioning, and modification of statements, highlighting joint decision-making. Finally, the RP occurs when participants reach a mutual agreement, choosing a shared statement that reflects the collective decision of the dyad. This final phase is defined by the convergence of individual preferences into a single, agreed-upon outcome. The criterion used here for classification incorporates both the semantic content of the communication and the underlying interactional structure of the negotiation process.

Based on semantic and temporal segmentation, the negotiation task was divided into three standardized phases across all dyads, each with an approximate duration of 60 s: IP, NCP, and RP. This structure was derived from a preliminary descriptive analysis conducted dyad-by-dyad on the recorded verbal exchanges, which confirmed the reliability and interpretative utility of this tripartite division based on recurrent linguistic markers and pragmatic transitions.

#### 2.2.2. Individual Differences Assessment

Following the main experiment, participants completed an assessment step (e.g., individual differences assessment) involving the administration of the following questionnaires: the GDMS ([50,51]; measures decision-making preferences across styles such as rational, intuitive, dependent, avoidant, and spontaneous), the MS ([52,53]; assesses maximization tendencies across dimensions such as alternative search, decision difficulty, and satisficing), the BIS/BAS ([54,55]; assesses sensitivity to punishment and reward, including BIS, BAS Drive, BAS Fun Seeking, and BAS Reward Responsiveness), and the BFI ([56]; captures personality traits across five dimensions: openness, conscientiousness, extraversion, agreeableness, and neuroticism).

### 2.3. EEG and Autonomic Data: Acquisition and Processing

Before negotiation task initiation, a 120 s baseline measurement was recorded to collect participants’ EEG and autonomic responses at rest. Specifically, EEG data were recorded during the baseline registration and during the negotiation task using a 16-channel DC amplifier (SYNAMPS system) integrated with NEUROSCAN 4.2 software, adhering to the 10/20 international system for electrode placement. A total of 15 Ag/AgCl electrodes (Fp1, Fp2, F3, F4, Fz, Cz, C3, C4, T7, T8, Pz, P3, P4, O1, O2) were positioned on the scalp, with the earlobes serving as reference electrodes. Additionally, two electrooculographic (EOG) electrodes were placed at the outer canthus of the left eye to monitor and exclude ocular artifacts. Prior to data collection, electrode impedance was kept below 5 kΩ to ensure optimal signal quality. EEG signals were digitized at a sampling rate of 1000 Hz, with a 50 Hz notch filter applied to remove power line interference. For offline processing, a 0.01–50 Hz IIR bandpass filter was used, and the continuous EEG data were segmented into two-second epochs. Visual inspection was employed to identify and exclude epochs contaminated by ocular, muscular, or movement-related artifacts. Artifact-free segments were then analyzed using fast Fourier transform (FFT) with a Hamming window, yielding power spectral density (PSD) estimates with a spectral resolution of 0.5 Hz. PSD values were computed for the standard EEG frequency bands: delta (0.5–3.5 Hz), theta (4–7.5 Hz), alpha (8–12.5 Hz), beta (13–30 Hz), and gamma (30.5–50 Hz). To assess task-induced modulation of EEG activity, PSD values from the negotiation phase were normalized to baseline resting-state values using the formula [Normalized PSD = (PSD_task_ − PSD_BL_)/PSD_BL_], where PSD_task_ represents task-induced power and PSD_BL_ represents baseline power. Three anatomically defined regions of interest (ROIs) were used for further analysis: frontal (ROI-F: Fp1, Fp2, F3, F4), temporo-central (ROI-TC: T7, T8, C3, C4), and parieto-occipital (ROI-PO: P3, P4, O1, O2) (Figure 1b). Within each ROI, the mean PSD across the corresponding electrodes was calculated, allowing for a detailed comparison of regional brain activity during the negotiation task relative to the resting-state baseline. The EuDist was calculated on PSD values that were normalized to baseline. This normalization ensured that Synth effects reflected task-induced modulations rather than interindividual baseline differences. Additionally, hemispheric lateralization was considered by separately analyzing the mean PSD for electrodes located in the left (e.g., Fp1, F3, T7, C3, P3, O1) and right (e.g., Fp2, F4, T8, C4, P4, O2) hemispheres within each ROI (Figure 1b). The EuDist was calculated as the square root of the sum of squared differences in baseline-normalized PSD values between Member 1 and Member 2 for each ROI and frequency band. Specifically, EuDist = √Σ(PSD_1_ − PSD_2_)^2^, where PSD values were averaged across electrodes. This scalar index captures interindividual divergence in spectral amplitude, with lower values indicating stronger alignment. While EuDist does not detect phase-locked Synth, it provides a robust and synthetic measure of dyadic similarity, well suited for highlighting global task-related modulations in shared neural activation patterns.

Autonomic activity was monitored using a wearable sensor (Biofeedback, 2000 xpert system, Schuhfried GmbH) placed on the second finger of the non-dominant hand. GRS was measured as skin conductance level and response, while cardiovascular activity (CVA), assessed via photoplethysmography, was quantified as heart rate and heart rate variability. EDA data was sampled at 40 Hz, and photoplethysmography data at 100 Hz. All signals were reviewed offline to exclude artifacts.

SCR was derived from SCL using a moving average approach. HR was calculated from Blood Volume per Pulse data. To compute HRV, inter-beat intervals (IBI) were derived from HR data.

### 2.4. Statistical Data Analyses

Before performing statistical analyses, it was confirmed that all 13 dyads reached an agreement within the three-minute negotiation task. Specifically, five agreed on Member 1’s initial sentence, six on Member 2’s, and two on a third, previously unselected sentence. As the agreement condition was met, all 13 dyads were included in the analyses.

The statistical data analyses conducted in this study comprised both EEG and autonomic data evaluations. EEG and autonomic measures were analyzed at the inter-brain level (Section 2.4.1) using Euclidean distance (EuDist; dissimilarity indices) to assess Synth in PSD across frequency bands (delta, theta, alpha, beta, gamma) and autonomic indices (SCL, SCR, HR, HRV). Accordingly, we refer to our findings as reflecting inter-brain similarity or Synth in spectral amplitude, in line with prior literature using amplitude-based metrics in social neuroscience.

Furthermore, a hierarchical cluster analysis using Ward’s method (Section 2.4.2) was conducted on HRV data.

#### 2.4.1. Synth Analysis

For the Synth analysis, EEG activity and autonomic measures were evaluated at the dyadic level to quantify the degree of coherence in EEG and autonomic activation between Member 1 and Member 2. For EEG data, the EuDist was calculated between the PSD values of each frequency band across corresponding lateralization and ROIs within each dyad. Similarly, the Euclidean distance was computed for autonomic measures, including HR, HRV, SCL, and SCR.

Prior to statistical analysis, the mean and standard deviation of speech times from the trigger point were calculated, with only negotiation turns considered and conversational pauses or non-negotiation utterances excluded. For each participant, speech duration was averaged across all negotiation-related turns, excluding any non-negotiation utterances or pauses. This method was applied to both participants, ensuring comparability of speech durations, as indicated by the homogeneity of the mean and standard deviation.

For EEG data, five repeated-measures ANOVAs considering lateralization (2: left and right hemispheres), ROI (3: ROI-F, ROI-TC, ROI-PO), and phase (3: IP, NCP, RP) as within-subject independent variables were carried out considering the EuDist for the five different frequency bands (delta, theta, alpha, beta, and gamma) as distinct dependent variables. In the present study, the EuDist index was adopted to quantify amplitude-based divergence in normalized spectral power between dyad members. This choice was motivated by our aim to examine macro-level similarity in dyadic neural engagement across task phases, rather than temporal coupling.

For autonomic data, four repeated-measures ANOVAs with phases (3: IP, NCP, RP) as within-subject independent variables were applied to the following EuDist autonomic indices considered as dependent variables: HR, HRV, SCL, and SCR.

To ensure robust statistical interpretation, degrees of freedom were adjusted using the Greenhouse–Geisser correction when sphericity assumptions were violated. Post hoc pairwise comparisons were conducted to decompose significant interactions, with Bonferroni correction applied to mitigate the risk of Type I errors due to multiple testing. Effect sizes for significant effects were reported using partial eta squared (η^2^), while statistical significance was defined at α = 0.05.

Prior to conducting the primary analyses, data distribution was assessed for normality by examining skewness and kurtosis values. Descriptive statistics, including means and standard errors, were calculated for each condition to facilitate result interpretation. All statistical analyses were performed using Jamovi (version 2.6.22; The Jamovi Project, 2022).

#### 2.4.2. Cluster Analysis

**Figure 2:**
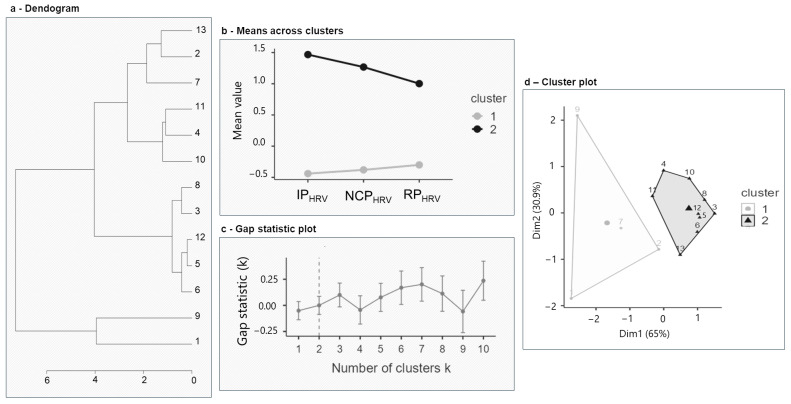
Cluster analysis results for HRV parameters. (**a**) A dendrogram representing the hierarchical clustering of the dataset. (**b**) Mean values of HRV parameters (IPHRV, NCPHRV, RPHRV) for the identified clusters. (**c**) A gap statistic plot indicating the optimal number of clusters. (**d**) A Principal Component Analysis (PCA) biplot showing the distribution of subjects in two-dimensional space (Dim1 and Dim2), with clusters represented by different symbols and convex hulls. Cluster 1 is depicted with circles, while Cluster 2 is represented with triangles.

A cluster analysis approach was used to identify latent groupings within a dataset based on the dyads’ physiological responses during the task. Both HR and HRV were initially examined; however, only HRV showed statistically significant phase-related modulation, and a repeated-measures ANOVA confirmed the absence of significant phase effects on HR (F_[2,24]_ = 0.673, *p* = 0.519). Therefore, the clustering analysis was conducted exclusively on the HRV metric, which served as an indicator of emotional reactivity. This decision ensured that the clustering captured the task-sensitive autonomic dynamics of the dyads. A hierarchical agglomerative cluster analysis was performed using Ward’s method with Euclidean distance as the similarity measure. The analysis involved 13 cases (dyads) and 3 variables for each phase: IP_HRV_, NCP_HRV_, and RP_HRV_. The optimal number of clusters was determined by visually inspecting the dendrogram (Figure 2a), which revealed hierarchical relationships between the cases and how they were progressively merged into clusters. A three-cluster solution was selected as the most robust and meaningful.

The initial hierarchical analysis identified three distinct clusters: Cluster 1 with 2 dyads (13 and 2), Cluster 2 with 6 dyads (7, 11, 4, 10, 8, and 3), and Cluster 3 with 5 dyads (12, 5, 6, 9, and 1). Following this, a k-means cluster analysis using the Hartigan–Wong algorithm was conducted to refine the initial groupings. This iterative method aimed to optimize cluster assignment by minimizing within-cluster variance, resulting in two distinct clusters based on HRV across different task phases (Cluster 1: 10 dyads; Cluster 2: 3 dyads).

Descriptive statistics (mean, standard deviation, minimum, and maximum) were computed for HRV within each cluster to further characterize the identified subgroups (within-cluster sum of squares for Cluster 1: $\sigma_{W_{1}}^{2}$ = 8.11; for Cluster 2: $\sigma_{W_{2}}^{2}$ = 9.30—between-cluster sum of squares was $\sigma_{B}^{2}$ = 18.58, indicating a total sum of squares of $\sigma_{T}^{2}$ = 36.00). The centroids of the clusters highlighted the mean HRV values across different task phases for each cluster (Cluster 1: IP_HRV =_ −0.440, NCP_HRV_ = −0.380, and RP_HRV_ = −0.301; Cluster 2: IP_HRV =_ 1.468, NCP_HRV_ = 1.268, and RP_HRV_ = 1.002). The plot of means across clusters (Figure 2b) visually represented the differences in HRV values between the two clusters across the task phases. Cluster 1 consistently showed lower HRV values, while Cluster 2 showed higher HRV values. The optimal number of clusters was further validated using the gap statistic method (Figure 2c). The gap statistic plot indicated that the optimal number of clusters was two, as it showed the highest gap statistic value at k = 2. The cluster plot (Figure 2d) provided a visual representation of the clustering solution in a two-dimensional space, showing the distinct separation between Cluster 1 and Cluster 2.

In addition to the gap statistic, the robustness of the clustering structure was supported by a silhouette coefficient of 0.48, indicating moderate internal consistency and separation between clusters. Furthermore, the bootstrap resampling procedure yielded a stability index of 0.92, suggesting high replicability of the two-cluster solution across subsampled iterations. These validation indices reinforce the interpretability and reliability of the final clustering outcome.

To evaluate the robustness of the clustering solution and explore potential differences between the identified clusters, a series of inferential analyses were conducted. One-way ANOVAs were performed using cluster (two levels: Cluster 1, Cluster 2) as the independent variable and various psychological scales as dependent variables. These included subscales from the GDMS, MS, BIS/BAS, and BFI. The significance threshold was set at α = 0.05. Where significant effects were identified, eta squared (η^2^) was reported as an effect size estimate, interpreted according to established conventions. All statistical analyses were performed using Jamovi (version 2.6.22; The Jamovi Project, 2022), and visualizations were generated to support the interpretation of results.

## 3. Results

### 3.1. EEG Synth Results

**Figure 3:**
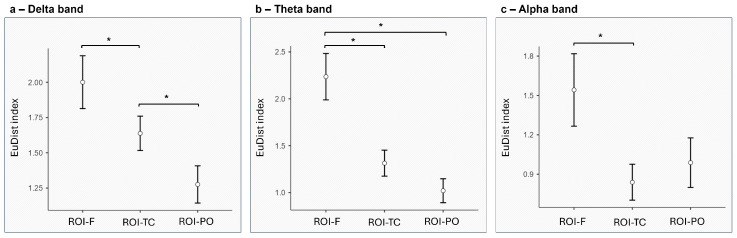
The EuDist index across different regions of interest (ROIs). Each panel represents a different frequency band: (**a**) delta, (**b**) theta, (**c**) alpha. The *y*-axis indicates the EuDist index, while the *x*-axis represents three brain regions: frontal (ROI-F), temporo-central (ROI-TC), and parieto-occipital (ROI-PO). Error bars represent Standard Error (SE). Asterisks (*) indicate statistically significant differences between ROIs.

*Delta Band.* A significant main effect was found for ROI (F_[2,24]_ = 6.563, *p* = 0.015, η^2^ = 0.049), with lower EuDist in ROI-PO compared to ROI-F (*p* = 0.036) and in ROI-PO compared to ROI-TC (*p* = 0.023) (Figure 3a).

*Theta Band.* A significant main effect was found for ROI (F_[2,24]_ = 10.388, *p* < 0.001, η^2^ = 0.146), with lower EuDist in ROI-TC compared to ROI-F (*p* = 0.045) and in ROI-PO compared to ROI-F (*p* = 0.006) (Figure 3b).

*Alpha Band.* A significant main effect was found for ROI (F_[2,24]_ = 8.550, *p* = 0.002, η^2^ = 0.073), with lower EuDist in ROI-TC compared to ROI-F (*p* = 0.045) (Figure 3c).

**Figure 4:**
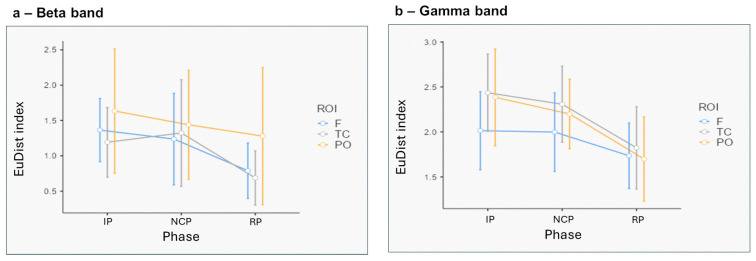
The EuDist index across task phases in the beta (**a**) and gamma (**b**) frequency bands, plotted separately for each region of interest (F = frontal; TC = temporo-central; PO = parieto-occipital). While descriptive trends suggest modest phase-related variations, no statistically significant effects or interactions were observed for either frequency band (all *p* > 0.05). Error bars represent standard deviations.

No significant differences were observed for the beta and gamma bands (Figure 4a,b).

### 3.2. Autonomic Synth Results

**Figure 5:**
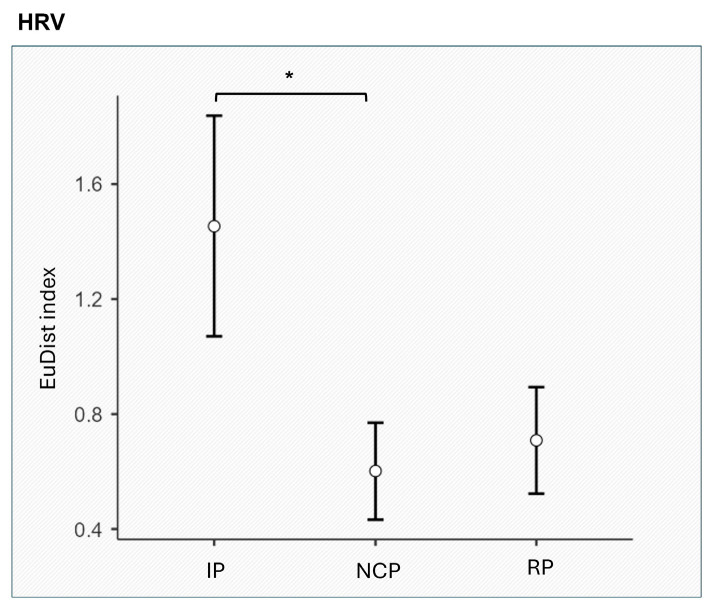
EuDist HRV index across phases (Initiation Phase IP, Negotiation Core Phase NCP, and Resolution Phase RP). Asterisks (*) indicate the statistically significant difference between the NCP and IP.

*HRV.* A significant main effect was found for phase (F_[2,24]_ = 5.353, *p* = 0.012, η^2^ = 0.147), with lower EuDist for the NCP compared to IP (*p* = 0.036) (Figure 5). No significant differences were observed for SCL, SCR, and HR.

### 3.3. Cluster Analysis Results

Although the sample size was limited, the between-cluster sum of squares (18.58) accounted for over 50% of the total variance (36.00), supporting a degree of separation. Moreover, the HRV centroids revealed stable differences across the three negotiation phases: Cluster 1 (N = 10) exhibited lower HRV values (IP = −0.440; NCP = −0.380; RP = −0.301), while Cluster 2 (N = 3) showed higher HRV across all phases (IP = 1.468; NCP = 1.268; RP = 1.002), suggesting distinct autonomic profiles.

**Figure 6:**
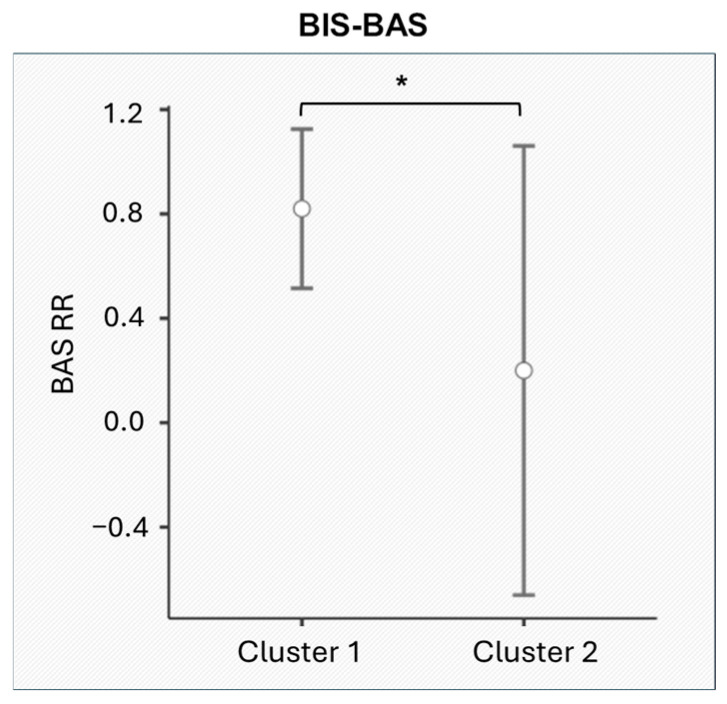
BAS Reward Responsiveness results. Asterisks (*) indicate the statistically significant difference between Cluster 1 and Cluster 2.

Significant differences were observed in the BAS Reward Responsiveness subscale of the BIS/BAS (F_[1,11]_ = 5.201, *p* = 0.043). Specifically, Cluster 1 exhibited significantly higher scores on BAS RR compared to Cluster 2 (Figure 6). No significant differences were found for other subscales, including the other BIS/BAS subscales and those from the GDMS, MS, and BFI.

## 4. Discussion

This study investigated the relationship between neural Synth and autonomic Synth in the context of dyadic exchanges centered on negotiation processes, with a specific emphasis on the social dynamics. The results underscore the significance of both neural Synth and autonomic modulation in facilitating effective interpersonal interactions.

Considering the EEG data, the observed significant effects in the delta, theta, and alpha bands across different ROIs suggest that inter-brain Synth during negotiation tasks primarily engages lower-frequency EEG oscillations. These findings align with theoretical frameworks emphasizing the fundamental role of low-frequency oscillations (e.g., delta and theta band) in neural communication and coordination [25,57].

Specifically, the delta band has been proposed to facilitate the integration of bottom-up sensory input with top-down cognitive control processes, allowing the brain to adaptively respond to external stimuli while maintaining an internal sense of emotional stability and cognitive focus [21,29]. This process is critical in environments that demand rapid shifts in attention and emotional regulation, as seen in high-stakes or emotionally charged interactions [58,59].

The lower EuDist in ROI-PO compared to ROI-F and ROI-TC suggests that the parieto-occipital areas are crucial for the integration of sensory inputs with internal representations. Indeed, the parieto-occipital cortex, which is involved in both visual–spatial processing and attentional control, plays a pivotal role in aligning sensory information with mental representations, thus supporting coherence in dyadic interactions [60]. This alignment function is essential for tasks that require mutual understanding and coordinated action, as it allows individuals to align their perceptual and cognitive experiences, enabling the construction of a shared mental model of the situation at hand. Moreover, theories such as the mirror neuron system (MSN) and theory of mind networks also provide further specifics to the interpretation of this first result. These theories suggest that regions like the parieto-occipital cortex are involved in the simulation of others’ experiences, allowing individuals to predict and align their mental states with those of their interaction partners [61,62]. These networks, which support the ability to understand others’ perspectives and intentions, are crucial for building consensus and maintaining effective communication in dyadic interactions [63].

Similarly, the results for theta band also show a lower EuDist in ROI-PO compared to ROI-F but also show a lower EuDist in ROI-TC compared to ROI-F. The significant finding of higher dissimilarity in theta-band activity within ROI-F compared to ROI-TC and ROI-PO highlights the differential roles of these brain areas in dyadic interactions. The frontal cortex, a key area for cognitive control and top-down regulatory functions [64], plays a central role in overseeing higher-level cognitive processes, such as conflict monitoring, error detection, and goal-directed behavior [65]. According to conflict monitoring theory, the anterior cingulate cortex detects conflicts during information processing, prompting necessary adjustments in cognitive control through the prefrontal cortex. These processes are inherently individualized, shaped by personal goals, contextual factors, and prior experiences [66,67]. Furthermore, error detection models suggest that such brain networks enhance learning by identifying mismatches between expected and actual outcomes [68,69]. Consequently, the Synth of these complex, internally driven cognitive processes between individuals is limited, as they operate within frameworks of unique personal goals, motivations, and adaptive responses to changing environments.

In contrast, the lower dissimilarity in ROI-TC and ROI-PO reflects a greater degree of shared processing. The temporo-central area, which encompasses areas like the STS and anterior temporal lobe, plays a central role in the social brain network [38,70]. This network supports processes such as intention attribution, emotional regulation, and the interpretation of social cues, facilitating alignment in dyads engaged in cooperative tasks [71]. Theta Synth in these regions likely supports a shared understanding and mutual regulation of emotional and social dynamics, which are essential for collaboration. Similarly, the parieto-occipital region, integral to the dorsal attention network (DAN), is heavily involved in coordinating joint attention and integrating sensory and spatial information [72,73]. Synth in this region may, as described for delta’s results reported above, reflect the alignment of attentional strategies, enhancing the dyad’s ability to process shared environmental cues and maintain cohesion throughout the task. These findings align with the functional specialization of neural networks, where posterior regions such as those in the DAN and social brain networks are naturally predisposed to Synth due to their roles in shared attention and social engagement. In contrast, frontal regions support task-specific, individualized regulation, which limits neural alignment. The lower dissimilarity in posterior regions suggests that these areas contribute to inter-brain coherence by integrating shared sensory and emotional information, whereas the frontal regions maintain a degree of functional independence necessary for task-specific executive control. This distinction may reflects the balance between synthronized and individualized processes that support effective collaboration.

Recent developments in dynamic network theory suggest that partial Synth states—often referred to as chimera states—may emerge during complex social interactions. These states are characterized by the coexistence of synthronized and desynthronized neural ensembles, reflecting functional differentiation across brain regions and systems. In our study, the observed regional dissociation—whereby posterior regions (ROI-TC and ROI-PO) exhibited lower inter-brain EuDist compared to frontal regions—could be interpreted as a manifestation of such hybrid states. Dynamic network analysis approaches have demonstrated that Synth is not uniformly distributed across time or space but may instead reflect transient and topographically specific alignments [74,75]. This interpretation aligns with our finding that social Synth engages lower-frequency oscillations in temporoparietal regions associated with shared attention and intention attribution, while frontal regions maintain a degree of functional independence necessary for individual executive control. These theoretical frameworks provide a valuable lens through which to interpret the flexible and regionally differentiated Synth observed during real-time negotiation.

Similarly, the alpha band results highlight higher EuDist in alpha-band activity within ROI-F compared to ROI-TC. Alpha oscillations are associated with inhibitory control processes that help filter task-irrelevant stimuli, enabling the allocation of attentional resources to relevant cues [33,76]. This mechanism, often described as gating by inhibition, allows for selective attention by suppressing distractors and enhancing task-related information processing [32]. Thus, a reduction in alpha power in ROI-TC during dyadic interactions indicates increased engagement of these regions, reflecting the suppression of irrelevant stimuli to prioritize collaborative goals. From a neural network perspective, the DAN works in conjunction with networks such as the DMN and the salience network (SN) to balance internal and external attentional demands [34]. Alpha band reductions in ROI-TC may suggest heightened engagement of the DAN in externally directed attention, while the DMN may be suppressed to minimize self-referential distractions. This dynamic ensures that individuals can maintain a focus on shared goals while remaining responsive to social and environmental cues. In fact, the observed reductions in alpha power further align with the predictive coding framework, which posits that the brain continuously generates predictions about incoming sensory information and updates these predictions based on feedback [77,78]. In social interactions, this involves predicting the behavior and intentions of others while suppressing irrelevant sensory input. Alpha oscillations facilitate this process by enhancing the signal-to-noise ratio [76,79], allowing individuals to focus on cues that confirm or refine their predictions about the interaction partner’s actions and intentions [30].

To sum up, the significant engagement of the delta, theta, and alpha bands across various ROIs highlights the foundational role of low-frequency oscillations in inter-brain Synth during this negotiation task.

The absence of significant differences in beta and gamma bands during inter-brain Synth suggests a predominant reliance on automatic, rather than effortful, cognitive processes. Indeed, neuroscientific evidence suggests that social Synth is driven by implicit processes [80]. Instead, higher-frequency oscillations are typically implicated in top-down executive functions, such as cognitive control, working memory, and complex problem-solving [23,24,81]. Such lack of significance in this context may indicate that inter-brain Synth concerns basic attentional and sensory integration rather than high-order cognitive functioning.

Considering the autonomic results, the significant phase-specific effects observed in HRV during the negotiation task highlight the role of autonomic regulation in facilitating effective social interactions. The finding of lower EuDist for HRV during the NCP compared to the IP suggests that dyads achieved a greater alignment in physiological responses as they engaged in collaborative decision-making processes. This physiological coherence reflects the dynamic relationship between autonomic nervous system activity and the cognitive and emotional demands of negotiation. Indeed, HRV is widely recognized as a biomarker of emotional regulation and social engagement [40]. The autonomic nervous system, particularly through parasympathetic modulation, supports adaptive responses to social and environmental challenges [39]. Lower EuDist during the NCP indicates increased alignment in autonomic states between members, potentially reflecting shared attentional focus and emotional attunement. This finding aligns with the polyvagal theory, which posits that vagal tone supports social engagement by fostering states of calmness and readiness for interaction [82]. In dyadic interactions, autonomic Synth in HRV may serve as a mechanism for building trust and facilitating cooperative behavior, as shared autonomic states promote mutual understanding and reduce interpersonal tension.

The absence of significant differences in SCL, SCR, and HR further underscores the specificity of HRV as a marker of social Synth. While SCL and SCR primarily reflect arousal-driven sympathetic activation [45], HRV captures the balance between sympathetic and parasympathetic influences [83,84,85], providing a more specific measure of emotional regulation and adaptive social behavior. The lack of significant effects for HR may suggests that overall arousal levels remained relatively stable across phases, while HRV modulation reflects the fine-tuned adjustments necessary for coherence.

The link between autonomic regulation and reward sensitivity in task engagement is further emphasized by the clustering analysis, that revealed distinct patterns of physiological and motivational dynamics within dyads. In Cluster 1, dyads, characterized by lower HRV and higher reward responsiveness, demonstrated an adaptive trajectory across task phases (see Figure 2b). Their heightened reward responsiveness may boost collective motivation, enhancing task engagement and collaboration. This aligns with the BAS framework, which links reward sensitivity to approach behaviors and cooperative interactions [86]. The theory of mutual reinforcement suggests that positive task outcomes create a feedback loop that strengthens motivational alignment [87]. This pattern supports theories of compensatory regulation [44,49], where dyads with initially lower regulatory capacity increase their HRV coherence over time, enhancing shared autonomic Synth. These dyads likely engage in a dynamic self-regulation process, consistent with models of cognitive flexibility and emotional resilience.

In contrast, in Cluster 2, dyads, with higher HRV and lower reward responsiveness, exhibited a higher baseline regulatory capacity, yet displayed a decline in HRV coherence as the task progressed. This suggests a progressive depletion of regulatory resources, in line with cognitive load theory, which posits that sustained effort under escalating demands can exceed regulatory capacity [88,89]. Their lower reward sensitivity suggests reliance on intrinsic or task-oriented motivation, which may limit sustained engagement as task demands intensify [90]. Moreover, this decline could reflect challenges in maintaining autonomic alignment, linked to the central executive network responsible for managing cognitive and emotional demands. While Cluster 2 dyads initially exhibit strong HRV coherence, their lower reward sensitivity may hinder motivation and collaboration under increasing cooperation demands.

These results are also consistent with the active inference framework, which conceptualizes cognition as a hierarchical process of predictive modeling and free-energy minimization. Within this perspective, inter-brain Synth in low-frequency oscillations may reflect the dynamic coupling of agents’ internal models, enabling them to jointly anticipate, interpret, and adjust to each other’s actions in real time. Delta and theta oscillations, in particular, are thought to support the integration of bottom-up sensory signals with top-down expectations, facilitating the recursive updating of shared representations. Alpha activity, by contrast, may contribute to the top-down gating of attention and the selection of relevant input, modulating the precision weighting of prediction errors during social interaction.

At the autonomic level, HRV coherence may indicate alignment of interoceptive predictions that underlie socio-emotional attunement. According to interoceptive extensions of active inference, the autonomic nervous system plays a pivotal role in encoding predictions about internal bodily states and regulating affective arousal in line with social affordances. Thus, the observed HRV Synth in our study may reflect a form of physiological model coupling that supports mutual adaptation and trust formation in collaborative contexts. Altogether, these patterns of neural and autonomic alignment can be understood as expressions of distributed predictive regulation, jointly supporting the construction of shared intentionality and the minimization of uncertainty in dyadic negotiation.

To conclude, the findings of this study highlight the critical role of delta, theta, and alpha oscillations in marking dyadic Synth during negotiation dynamics. Additionally, higher HRV coherence was associated with more adaptive negotiation outcomes, suggesting a robust link between autonomic regulation and interpersonal Synth. These results indicate that both neural and autonomic Synth serve as biomarkers of successful negotiation dynamics, influenced by regulatory mechanisms and approach motivation tendencies.

However, several methodological limitations must be acknowledged.

A major limitation of the present study concerns the relatively small sample size (13 dyads), which may constrain statistical power and increase the risk of Type II errors, particularly in exploratory procedures such as cluster analysis. Nonetheless, the sample size is comparable to previous EEG hyperscanning studies and, according to a priori power analysis (f = 0.38, α = 0.05, power = 0.95), meets the minimum threshold for detecting within-subject effects in a repeated-measures design involving 26 participants. Still, subgroup comparisons should be interpreted as preliminary and hypothesis-generating rather than conclusive. Larger and more heterogeneous samples will be required in future studies to improve the generalizability and robustness of the observed effects.

Moreover, the study’s focus on university students may limit the generalizability of the findings to broader populations, as cognitive strategies and personality traits can vary significantly across different age groups and educational backgrounds. Future research should consider more diverse participant samples to enhance the applicability of these results.

Furthermore, while our use of Euclidean distance allowed us to assess global alignment in EEG spectral features across dyads, this approach does not capture the temporal precision of phase-based neural coordination. Our focus was on condition-induced modulations in dyadic neural similarity, rather than transient Synth dynamics. However, future research may benefit from integrating phase-coupling measures, such as Phase Locking Value (PLV), to explore the real-time dynamics of inter-brain connectivity during negotiation.

Moreover, the present study adopted a region-based approach to inter-brain Synth, focusing on macro-anatomical ROIs rather than lateralized subdivisions. While this allowed us to capture global patterns of alignment between dyad members, it precluded the analysis of potential hemispheric asymmetries. Future studies may explore lateralized inter-brain dynamics—such as left vs. right frontal coupling—which could yield more nuanced interpretations of social Synth processes, particularly in relation to affective valence and communicative roles.

One further limitation of the current study is the absence of a control condition involving parallel but non-interactive decision-making. Without such a comparison, it remains unclear whether the observed Synth effects are uniquely attributable to interpersonal negotiation or reflect more general cognitive engagement. Future studies should implement non-dyadic or non-cooperative control paradigms to better isolate the specific contribution of social interaction.

Concluding, these findings presented in this study should be interpreted with caution due to the small sample and the inherently exploratory nature of the cluster analysis. Although the internal structure of the clusters appears consistent, further validation in larger samples is necessary before drawing robust conclusions on motivational-autonomic coupling during negotiation. Looking beyond these limitations, the implications of these findings are significant for real-world applications. By understanding the neural and physiological mechanism of successful negotiations, we can develop strategies to enhance interpersonal Synth in various settings. This knowledge can inform the design of interventions aimed at improving communication and collaboration in professional environments, conflict resolution scenarios, and cooperative decision-making processes.

## Data Availability

The data presented in this study are available on request from the corresponding author due to ethical reasons for sensitive personal data protection (requests will be evaluated according to the GDPR-Reg. UE 2016/679 and its ethical guidelines).
